# Deconvolution of tumor composition using partially available DNA methylation data

**DOI:** 10.1186/s12859-022-04893-7

**Published:** 2022-08-24

**Authors:** Dingqin He, Ming Chen, Wenjuan Wang, Chunhui Song, Yufang Qin

**Affiliations:** 1 College of Information Technology, Shanghai Ocean University, Hucheng Ring Road, Shanghai, China; 2 Key Laboratory of Fisheries Information Ministry of Agriculture, Shanghai, China

**Keywords:** Cell population proportions, Tumor heterogeneity, Immunotherapy, DNA methylation data, Non-negative matrix factorization

## Abstract

**Background:**

Deciphering proportions of constitutional cell types in tumor tissues is a crucial step for the analysis of tumor heterogeneity and the prediction of response to immunotherapy. In the process of measuring cell population proportions, traditional experimental methods have been greatly hampered by the cost and extensive dropout events. At present, the public availability of large amounts of DNA methylation data makes it possible to use computational methods to predict proportions.

**Results:**

In this paper, we proposed PRMeth, a method to deconvolve tumor mixtures using partially available DNA methylation data. By adopting an iteratively optimized non-negative matrix factorization framework, PRMeth took DNA methylation profiles of a portion of the cell types in the tissue mixtures (including blood and solid tumors) as input to estimate the proportions of all cell types as well as the methylation profiles of unknown cell types simultaneously. We compared PRMeth with five different methods through three benchmark datasets and the results show that PRMeth could infer the proportions of all cell types and recover the methylation profiles of unknown cell types effectively. Then, applying PRMeth to four types of tumors from The Cancer Genome Atlas (TCGA) database, we found that the immune cell proportions estimated by PRMeth were largely consistent with previous studies and met biological significance.

**Conclusions:**

Our method can circumvent the difficulty of obtaining complete DNA methylation reference data and obtain satisfactory deconvolution accuracy, which will be conducive to exploring the new directions of cancer immunotherapy. PRMeth is implemented in R and is freely available from GitHub (https://github.com/hedingqin/PRMeth).

**Supplementary Information:**

The online version contains supplementary material available at 10.1186/s12859-022-04893-7.

## Background

Intra-tumor heterogeneity is formed by the dynamic interactions of different tumor cell populations (or subclones), infiltrating immune cells, and stromal cells in the tumor microenvironment [1–4]. Studies have shown that intra-tumor heterogeneity is closely related to clinical prognoses such as tumor growth, metastasis, recurrence, and drug resistance [5]. The tumor heterogeneity can be measured by the number of cell populations in tumor tissues, their molecular profiles, and their proportions. Specifically, cell type proportion prediction is an important task for multi-omic data analysis or clinical studies. For example, accounting for cell type proportions is proven to be helpful for Epigenome-Wide Association Study (EWAS) [6], and the composition of infiltrating immune cells in tumor tissues is predictive of the response to checkpoint inhibitor immunotherapy [7].

Currently, experimental techniques including flow cytometry and single-cell techniques such as Drop-seq [8], 10X Genomics, and sci-RNA-seq [9] have been used to study cellular components in complex tissues, but they are costly [10] and sensitive to technical changes during cell isolation. Thus, in recent years, computational estimation of cellular components using gene expression or DNA methylation data has become a hot topic in computational biology [10–27]. Compared to gene expression, DNA methylation has the advantage of being more stable [28], highly cell-type specific [29], and easier to measure in formalin-fixed paraffin-embedded (FFPE) tissues [30]. As a result, DNA methylation is more suitable for studying cellular components. Currently, the methods based on DNA methylation can be broadly classified into two categories: reference-based methods and reference-free methods. Among the reference-based methods, Houseman et al. [11] proposed a linear regression method (QP) based on DNA methylation, which uses quadratic programming to ensure that the regression coefficients are non-negative. Teschendorff et al. [16] developed EpiDISH, which uses non-constrained weighted linear regression rather than linear regression to reduce the weights of data points with large residuals. Altboum et al. proposed DCQ [13], which modifies the deconvolution approach into a regularized regression model to reduce the number of model parameters. Inspired by the success of CIBERSORT [14] in gene expression decomposition, Chakravarthy et al. [10] analyzed the cell type composition of complex mixtures using support vector regression based on DNA methylation data and obtained more accurate estimates. The latest reference method based on DNA methylation is Emeth [21], which uses a mixture distribution based on ICeD-T [20] to identify CpG sites whose DNA methylation in tumor samples is inconsistent with the reference methylation profiles and to reduce the contribution of these aberrant sites in cell type abundance estimation.

The general limitation of the above reference methods is that they require DNA methylation profiles of specific cell types as input, but in practice, it is difficult to obtain DNA methylation profiles of all cellular components in tumor tissues [31]. To overcome this limitation, many researchers have developed reference-free methods. For example, James et al. [22] proposed a combination (FAST-LMM-eWasher) of linear mixed models and principal components to correct the composition of cell types automatically. Houseman et al. [23] applied an iterative quadratic programming framework (RF) to DNA methylation for cell type analysis. Motivated by previous research, Lutsik et al. [26] developed MeDeCom by combining constrained non-negative matrix factorization with a new biologically relevant regularization function. Such methods do not rely on reference information and aim to estimate molecular profiles and proportions of all cell types simultaneously, unfortunately, their prediction accuracies are far from satisfactory. However, in real clinical practice, gene expression or DNA methylation is often available for only a small fraction of cell types, and reference information for the remaining cell types is unknown. To overcome these limitations, easily available data for a portion of cell types in a tumor mixture can be used as a reference to deconvolute the entire tumor mixture.

In this paper, we proposed a method for *p*artially–*r*eference cell type decomposition using DNA *meth*ylation data (PRMeth). PRMeth used an iteratively optimized non-negative matrix factorization framework, which took DNA methylation profiles of a portion of the cell types in the tissue mixtures (including blood and solid tumors) as input to estimate the proportions of all cell types as well as the methylation profiles of unknown cell types simultaneously. Based on three benchmark datasets, we compared PRMeth with five different methods (i.e., Reference-Free (RF) [23], Quadratic Programming (QP) [11], CIBERSORT (CBS) [14], Digital cell quantification (DCQ) [13], and Epigenetic Dissection of Intra-Sample Heterogeneity (EpiDISH) [16]). The results showed that PRMeth outperformed the other five methods. PRMeth was then applied to four types of tumors from The Cancer Genome Atlas (TCGA) [32] database, i.e., skin cutaneous melanoma (SKCM), invasive breast carcinoma (BRCA), acute myeloid leukemia (LAML), and thymoma (THYM). The experimental results revealed that immune cell proportions estimated by PRMeth were in good agreement with previous studies and PRMeth could provide new insights into tumor heterogeneity and immunotherapy.

## Methods

### Simulation data

The simulation dataset was constructed from five immune cells (including neutrophils, CD4 + T cells, CD8 + T cells, natural killer cells (NK), CD19 + B cells) (GSE88824), one non-small cell lung cancer cell (A549), and one normal human bronchial epithelial cell (NHBEC) (GSE92843) available from the Gene Expression Omnibus (GEO) [33]. To obtain the methylation profiles of the cell types, we loaded their respective IDAT files using the *champ.load* (ChAMP package in R) and filtered out 79,818 probes with a detection *p* value > 0.01, a beadcount < 3 in at least 5% of samples, non-CPGs, SNPs, MultiHit, and locating on X, Y chromosome. Then, the filtered data were normalized by the *champ.norm* and their batch effects were eliminated by the *champ.runCombat*. Finally, we were able to obtain the methylation profiles for seven different cell types (recorded as base profiles).

Next, the base profiles were employed to generate the methylation profiles of non-small cell lung cancer (NSCLC) samples with different cell type proportions and levels of noise. In the first step, we randomly generated the proportions of all cell types for each NSCLC sample based on the Dirichlet distribution. In detail, the proportions of A549 cell, NHBEC, and immune cells are 60%, 10%, and 30%, respectively. These proportions are in accordance with the true proportions of the cell types found in NSCLC samples [25]. In the second step, we generated methylation profiles of the cell types with different levels of noise from an independent beta distribution with mean and variance inferred from the base profiles (see Results for details). In the third step, the methylation profiles of the cell types with different noise levels were linearly combined according to the above ratios as the methylation profiles of NSCLC samples. In the end, the methylation profiles of 100 NSCLC samples were obtained. We used the Dirichlet distribution to generate proportions of cell types 20 times randomly and then obtained 20 simulation datasets at each noise level. The 20 simulation datasets are used to validate the performance of the proposed method PRMeth.

### Real data obtained from experiments

Besides the simulation dataset, we also applied our method to the following three datasets. In the first dataset, the methylation profiles of 100 mixture samples, the methylation profiles of seven types of immune cells (including CD4 + T cells, CD8 + T cells, monocytes, B cells, NK cells, neutrophils, and T regulatory cells) constituting mixture samples, and the proportions of all cell types for each sample were provided by Zhang et al. [21]. This dataset is referred to as the Zhang dataset in this paper.

In the second dataset, the methylation profiles of six whole blood samples and their constitutional cell types (including CD4 + T cells, CD8 + T cells, monocytes, B cells, NK cells, neutrophils, and eosinophils) were obtained from Chakravarthy et al. [10] via the GEO accession number GSE35069, and the proportions of each cell type were measured by flow cytometry as provided by the authors [34].

In the third dataset, the methylation profiles of skin cutaneous melanoma (SKCM), invasive breast carcinoma (BRCA), acute myeloid leukemia (LAML), and thymoma (THYM) samples were downloaded from the TCGA database. To facilitate the comparison, 100 tumor samples were randomly selected for each cancer type. As the reference for deconvolution, the methylation profiles of seven immune cells (including monocytes, dendritic cells, macrophages, eosinophils, naive T cells, CD8 + T cells, and NK cells) were obtained from Arneson et al. [35] via the GEO accession numbers GSE35069, GSE59250, and GSE71837. Meanwhile, the batch effects between the methylation profiles of tumor samples and those of immune cell types were eliminated by the *ComBat* function in *sva* package of R.

### PRMeth model construction

**Fig. 1:**
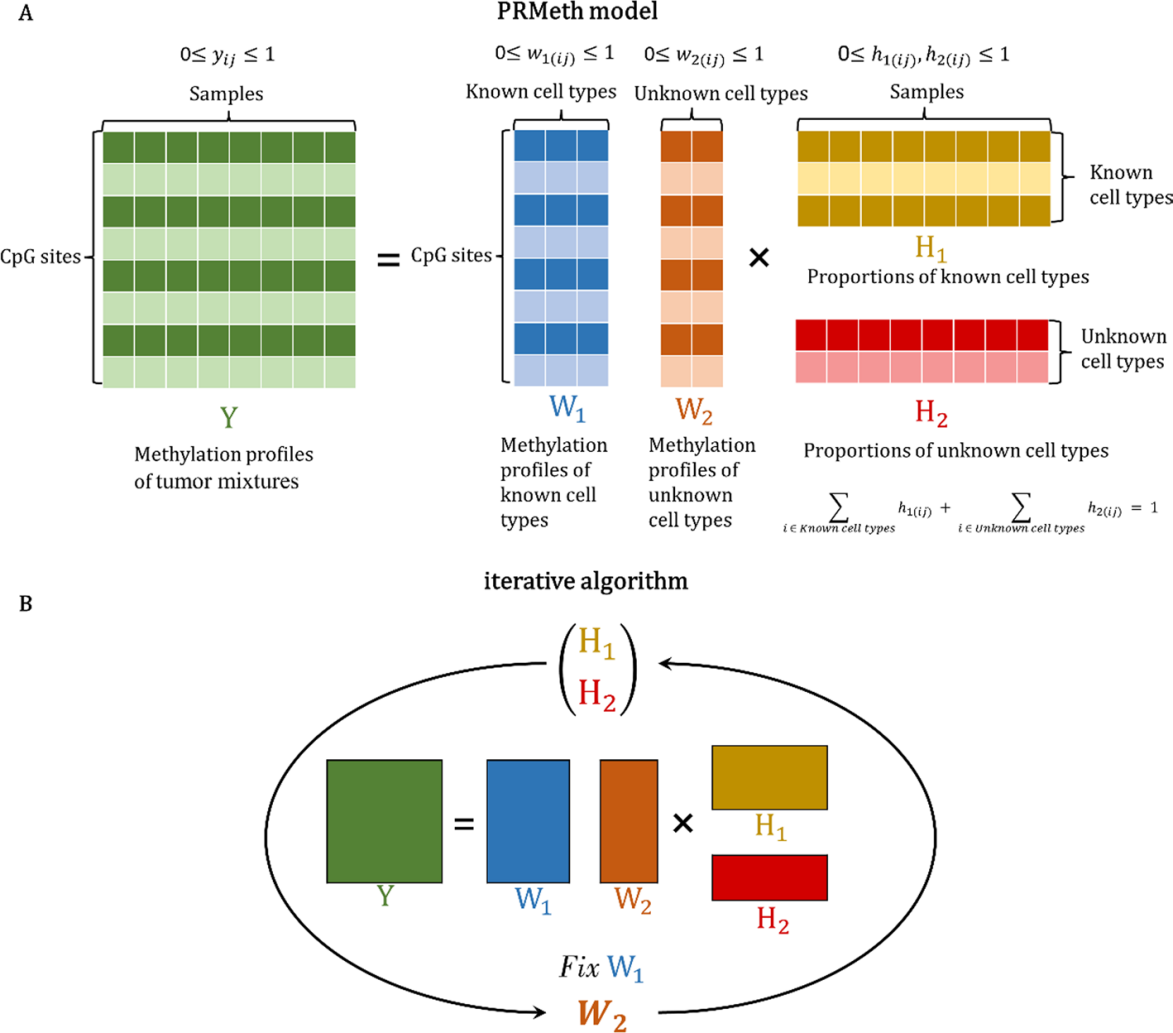
The framework of the PRMeth model. **A** The non-negative matrix factorization scheme of the PRMeth model. **B** The iterative algorithm of the PRMeth model

The framework of PRMeth is illustrated in Fig. 1. It is assumed that the methylation profiles of tumor tissues are mixture signals from their constitutional cell types, where only a part of them have available methylation profiles. We proposed a non-negative matrix factorization scheme (Fig. 1A) and an iterative algorithm (Fig. 1B) to estimate the proportions of all cell types and the methylation profiles of unknown cell types simultaneously.

We denote $Y\in {R}_{+}^{m\times n}$ as the methylation profiles of *m* CpG sites in *n* tumor mixtures. Suppose that the tumor mixtures are made up of $K$ cell types with a certain proportion. According to the deconvolution model:

1 $$Y=\left({{W}_{1,}W}_{2}\right)\left(\genfrac{}{}{0pt}{}{{H}_{1}}{{H}_{2}}\right)+\varepsilon$$

where ${W}_{1}\in {R}_{+}^{m\times {K}_{1}}$, ${W}_{2}\in {R}_{+}^{m\times {K}_{2}}$ denote the methylation profiles of ${K}_{1}$ known cell types and ${K}_{2}$ unknown cell types ($K={K}_{1}+{K}_{2}$), ${H}_{1}\in {R}_{+}^{{K}_{1}\times n}$ and ${H}_{2}\in {R}_{+}^{{K}_{2}\times n}$ denote the proportions of known and unknown cell types, respectively. $\varepsilon$ is an $m\times n$ error matrix. Observing that ${y}_{ij}\in Y$, ${w}_{1\left(ij\right)}\in {W}_{1}$ and ${w}_{2(ij)}\in {W}_{2}$ represent the DNA methylation level (i.e., beta value) of a CpG site, then $0\le {{y}_{ij},h}_{1\left(ij\right)},{h}_{2\left(ij\right)}\le 1$. And the proportions ${h}_{1\left(ij\right)},{ h}_{2\left(ij\right)}$ of $K$ cell types in a mixture satisfy $0\le {h}_{1\left(ij\right)},{h}_{2\left(ij\right)}\le 1$ and $\sum_{i=1}^{{K}_{1}}{h}_{1\left(ij\right)} + \sum_{i={K}_{1}+1}^{{K}_{2}}{h}_{2\left(ij\right)}= 1$.

In this model, the methylation profiles $Y$ of the mixtures and the methylation profiles ${W}_{1}$ of the partial cell types were known, and we aimed to estimate the proportions $\left(\genfrac{}{}{0pt}{}{{H}_{1}}{{H}_{2}}\right)$ of all cell types and the methylation profiles ${W}_{2}$ of unknown cell types, which could be obtained by solving for the minimization error sum of squares, thus transforming Eq. (1) into:

$$\left(\widehat{{W}_{2}},\widehat{{H}_{1}},\widehat{{H}_{2}}\right)={argmin}_{{W}_{2},{H}_{1},{H}_{2}}{\Vert Y-\left({{W}_{1, }W}_{2}\right)\left(\genfrac{}{}{0pt}{}{{H}_{1}}{{H}_{2}}\right)\Vert }_{F}^{2}$$

2 $$={argmin}_{{W}_{2},{H}_{1},{H}_{2}}{\Vert Y-{W}_{1}{H}_{1}-{W}_{2}{H}_{2}\Vert }_{F}^{2}$$

3 $$\mathrm{s}.\mathrm{t}. 0\le {w}_{2\left(ij\right)},{h}_{1\left(ij\right)},{h}_{2\left(ij\right)}\le 1$$

4 $$\sum_{i=1}^{{K}_{1}}{h}_{1\left(ij\right)} + \sum_{i={K}_{1}+1}^{{K}_{2}}{h}_{2\left(ij\right)}= 1$$

where $||\cdot {||}_{F}^{2}$ denotes the *Frobenius* norm.

Next, an iterative algorithm was used to estimate $\left(\genfrac{}{}{0pt}{}{{H}_{1}}{{H}_{2}}\right)$ and ${W}_{2}$. As shown in Fig. 1B, we fixed the obtained partial reference data ${W}_{1}$ and iterated the values of $\left(\genfrac{}{}{0pt}{}{{H}_{1}}{{H}_{2}}\right)$ and ${W}_{2}$ continuously by Eq. (2) to calculate their final results. The detailed algorithm flow is as follows:
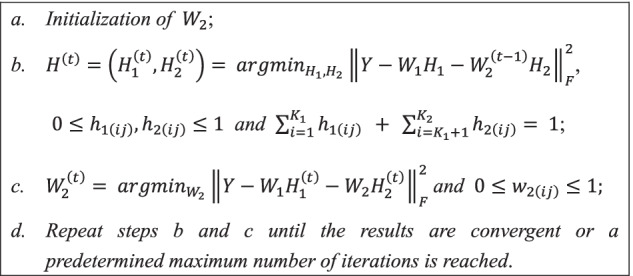
 where $t$ is the number of iterations. In step *a*, we employed the RPMM [36] algorithm to initialize the methylation profiles ${W}_{2}$ of unknown cell types. In detail, RPMM is a clustering algorithm that clusters the methylation profiles $Y$ of tumor samples into ${K}_{2}$ clusters by the *binary* distance formula and takes the clustering centers as the initial value of ${W}_{2}$. Furthermore, we compared RPMM with six initialization approaches including five different clustering algorithms (i.e., canberra, euclidean, manhattan, maximum, and minkowski) and a random generation algorithm (random). As shown in Additional file 1: Figure S1, there were no significant differences between the seven methods, but RPMM outperformed the other approaches in estimating proportions on the simulation dataset.

For PRMeth, if profiles of all constitutional cell types are available (i.e., ${K}_{1}=K$), it is actually the QP method. On the contrary, if none of the constitutional cell types is known ($\mathrm{i}.\mathrm{e}., {K}_{1}=0$), the PRMeth method turns to the RF method. Therefore, the PRMeth method is a more general framework that includes the reference-based and reference-free methods as two special cases.

### CpG site selection

The total number of CpG sites in the human genome is very huge. To reduce the potential noise and improve the computational efficiency, we selected CpG sites with high methylation variation in tumor samples by the coefficient of variation (${c}_{v}$) as follows:

5 $${c}_{v}=\frac{\sigma }{\mu }$$

where $\sigma$ and $\mu$ denote the standard deviation and mean of a CpG site in $Y$, respectively. We sorted these sites according to ${c}_{v}$ and then selected the top *n* with the highest ${c}_{v}$ values as input features.

### Cell type number prediction

In our method, the number $K$ of cell types in tumor mixtures needs to be specified. The Bayesian information criterion (BIC) [37] is an important measure of model superiority that can give the optimal number of parameters in the model. Therefore, BIC was selected to identify $K$ in the tumor mixtures. Furthermore, in order to weaken the penalty, a penalty factor $\mathrm{was introduced}$. $\lambda \_BIC$ is defined by the formula:

6 $$\lambda \_BIC=N\mathit{ln}\left(\frac{SSR}{N}\right)+\lambda P\mathit{ln}\left(N\right)$$

where $N$ denotes the sample size, $P$ denotes the number of model parameters, $SSR$ denotes the residual sum of squares between the true and estimated methylation profiles of the tumor mixtures, and $\lambda$ denotes the penalty factor, whose size is restricted to $(0, 1)$. In the PRMeth model, $N=n\times m$ as well as $P=K\left(n+m\right)-{nK}_{1}$, where $n$, $m$, $K$ and ${K}_{1}$ denote the number of tumor mixtures, the number of CpG sites, the total number of cell types, and the number of known cell types, respectively. Different $K$ values correspond to different $\lambda \_BIC$ values, and the $K$ value corresponding to the smallest $\lambda \_BIC$ value is the optimal number of cell types for the tumor mixtures.

## Results

### Research design

The five methods, i.e., QP, DCQ, EpiDISH, RF, and CBS, are state-of-the-art methods for the DNA methylation deconvolution task. Among them, RF, QP, DCQ, and EpiDISH used the linear model and CBS used the most popular non-linear model (support vector regression). The two models were also adopted by the other deconvolution methods introduced in the Background section, so we compared PRMeth with the five methods.

1. RF [23], a reference-free method for solving cell type proportions and cell type methylation profiles using iterative quadratic programming;
2. QP [11], a reference-based method for solving cell type proportions using quadratic programming;
3. CBS [14], a reference-based method for inferring the proportions of tumor-infiltrating immune cells using support vector regression;
4. DCQ [13], a reference-based method for inferring the global dynamics of the number of immune cells in complex tissues using elastic net regularization.
5. EpiDISH [16], a reference-based method for estimating cell type proportions using non-constrained weighted linear regression.

The mean absolute error (MAE) and Pearson correlation coefficient (PCC) were used to evaluate the performance of different methods. In detail, MAE measures the mean absolute error between the estimated and true values of cell type proportions or cell type methylation profiles, and PCC quantifies the correlation coefficient between the estimated and true values of cell type proportions or cell type methylation profiles, with values ranging from $[-1, 1]$.

### Determination of the number of cell types

The number of cell types should be specified first for PRMeth. However, it is not a trivial task since we are infeasible to know the exact number without a single-cell sequencing experiment. We here determined the number $K$ of cell types in mixture samples using $\lambda \_BIC$, a modified Bayesian information criterion (see Methods for details). Assuming that the methylation profiles $Y$ of tumor mixtures and the methylation profiles ${W}_{1}$ of ${K}_{1}$ cell types are known, penalty factor $\lambda$ is taken as $\mathrm{0.1,0.2},\dots ,0.9$, and $K$ is chosen as ${K}_{1}+1,{K}_{1}+2,\dots ,{K}_{1}+k$, where $k\le 30$. All $\lambda$ and $K$ were traversed to calculate their corresponding $\lambda \_BIC$ values. The optimal number of cell types was determined as the $K$ with the smallest $\lambda \_BIC$ value.

**Fig. 2:**
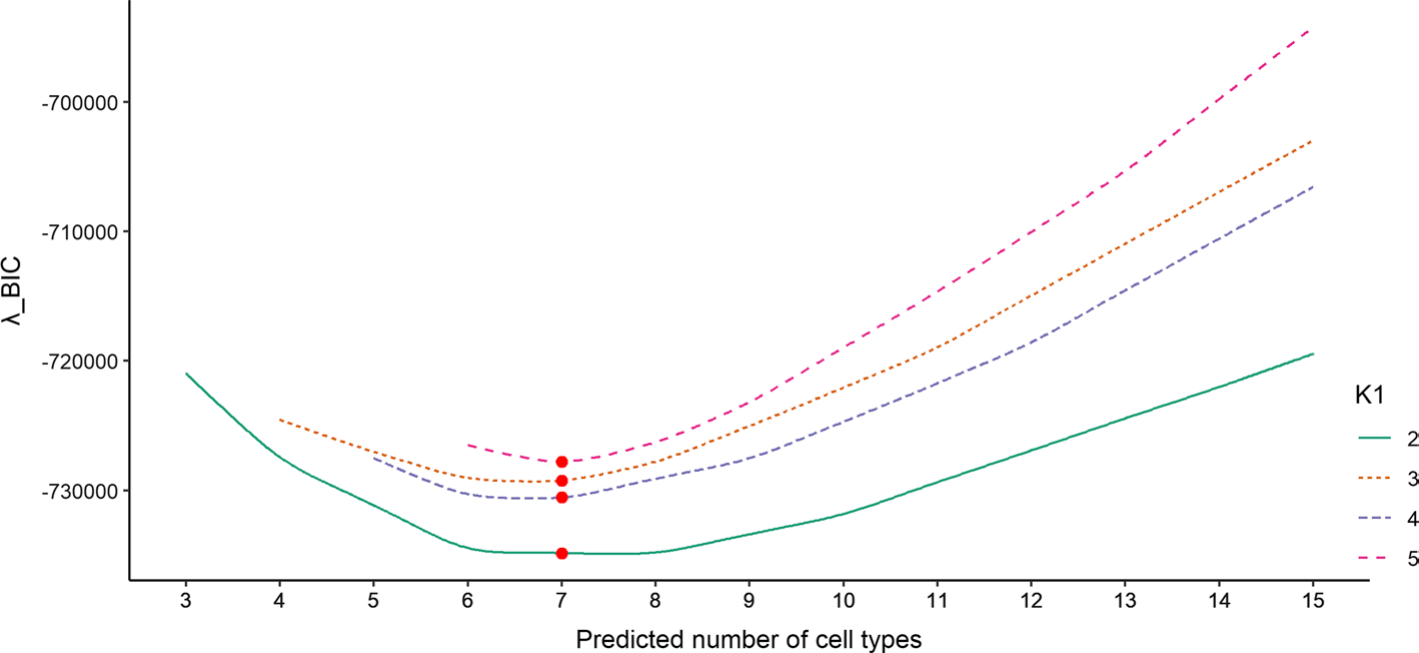
Accuracy of $\lambda \_BIC$ in predicting the total number of cell types. $\lambda \_BIC$ values when the true total number of cell types is seven, but only 2, 3, 4, or 5 cell types are known and their corresponding penalty factor is 0.3, 0.4, 0.4, or 0.5

$\lambda \_BIC$ was tested on the Zhang dataset with in total 7 cell types by setting ${K}_{1}$ as 2, 3, 4, and 5, respectively. It was observed that the smallest $\lambda \_BIC$ values corresponded to $\lambda =0.3, 0.4, 0.4, 0.5$, and $K=7$ for all ${K}_{1}$. When $\lambda$ was fixed as $0.3, 0.4, 0.4,$ or $0.5$, we plotted the $\lambda \_BIC$ values with $K$ as shown in Fig. 2. As expected, all $\lambda \_BIC$ values decreased first and then increased with the increase of $K$, and PRMeth could successfully predict the correct number ($K=7$) of cell types in all scenarios.

### Evaluation of different methods using simulation data

**Fig. 3:**
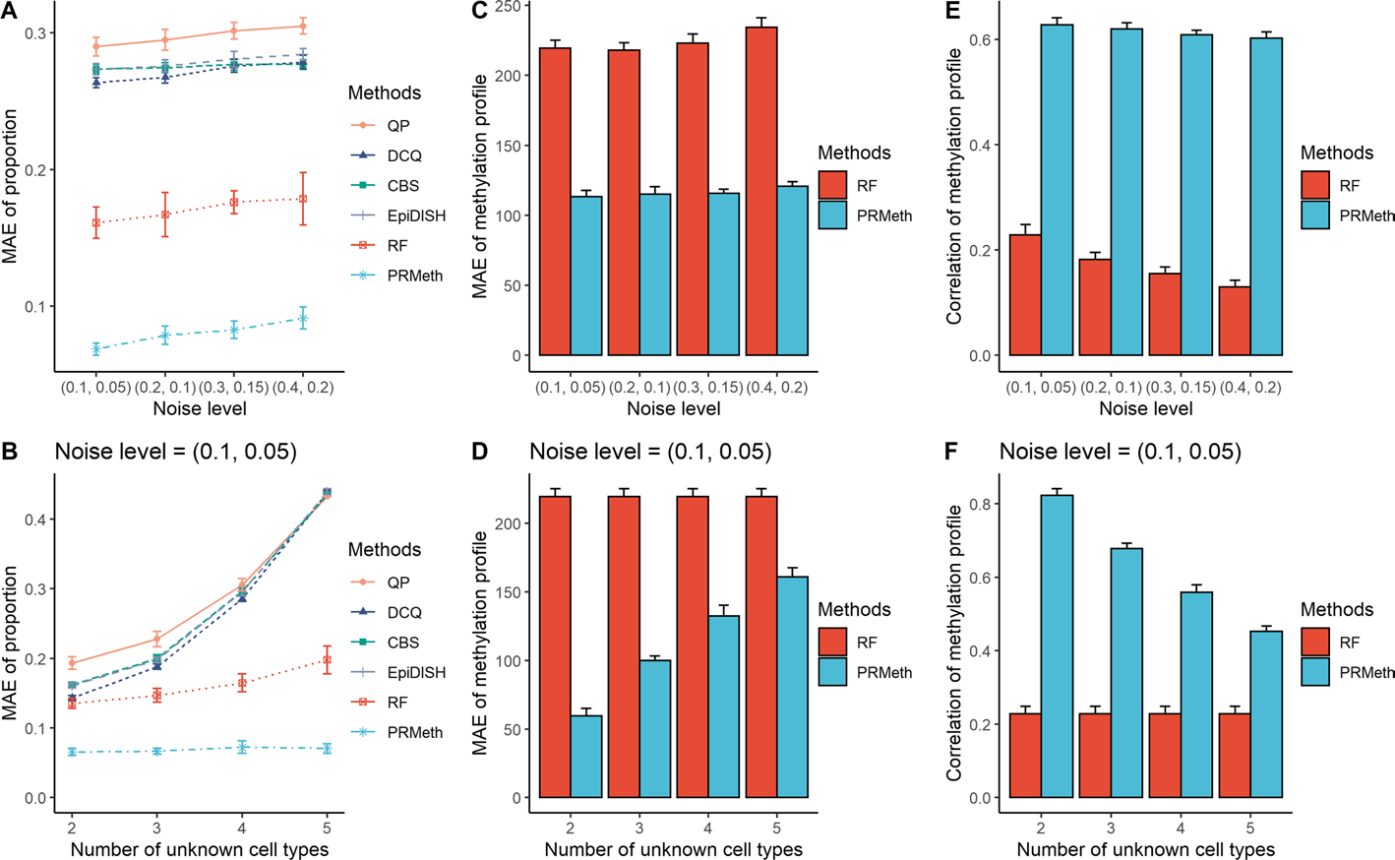
Accuracy of cell type proportions and cell type methylation profiles estimated by different methods. **A**, **B** The mean absolute error between the true and predicted cell type proportions obtained by six methods from the simulation dataset with different noise levels (**A**) or different unknown cell type numbers (**B**). **C**–**F** The mean absolute errors (**C**, **D**) and Pearson correlation coefficients (**E**, **F**) between true and predicted cell type methylation profiles obtained by PRMeth and RF at different noise levels or different unknown cell type numbers. All simulations were repeated 20 times

After successfully determining the total number of cell types, we next evaluated the estimation accuracy of PRMeth on the simulation dataset. First, the top 1000 CpG sites with the highest coefficient of variation (${c}_{v}$) were selected as the input for six methods. Then, we calculated the mean absolute error (MAE) between the true and predicted proportions of available cell types for each method at different noise levels. Here, the random noise was generated by a beta distribution whose mean is the methylation level of each site for each cell type in the base profiles and whose variance is a certain percentage of the maximum variance (i.e., $mean*(1-mean)$) calculated by the above mean. In detail, we took 10%, 20%, 30%, and 40% of the maximum variance when processing lung cancer cell types and 5%, 10%, 15%, and 20% for normal cell types. As shown in Fig. 3A, the MAE of all six methods increased with the increase of the noise level. Compared to other methods, PRMeth consistently obtained the lowest bias and relatively stable results at all noise levels. When the noise level was (0.1, 0.05), we evaluated the performance of PRMeth in estimating the proportions of cell types at different numbers (${K}_{2}=2, 3, 4, 5$) of unknown cell types. It is shown that PRMeth always obtained the lowest and most stable bias, however, the MAE of the remaining methods all gradually increased with the increasing number of unknown cell types (Fig. 3B). For the three remaining noise levels (0.2, 0.1), (0.3, 0.15), and (0.4, 0.2), PRMeth performed similarly well (Additional file 1: Figure S2).

In addition to proportion prediction, PRMeth (as well as RF) can also infer the methylation profiles of cell types. Figure 3C–F show the MAE and Pearson correlation coefficient (PCC) between the true and predicted cell type methylation profiles calculated by the two methods at different noise levels or different numbers of unknown cell types. At all noise levels, PRMeth achieved consistently higher accuracy (Fig. 3C) and correlation (Fig. 3E) compared to RF. Furthermore, when the noise level was (0.1, 0.05), the MAE of PRMeth gradually increased but remained lower than RF (Fig. 3D) and its PCC decreased gradually but remained higher than RF (Fig. 3F) with the increasing number of unknown cell types. PRMeth exhibited the same results as Fig. 3D, F compared to the reference-free method at the three remaining noise levels (Additional file 1: Figure S3).

We also evaluated the computational performance of these six methods. As shown in Additional file 1: Table S1, executing 20 times at 100 samples and 1000 CpG sites, both the running time and memory usage of PRMeth is a little higher than the other methods. This is because many iterations are required to reach the optimal solution. In addition, we analyzed the running time and memory usage of PRMeth when the number of samples and features gradually increased. This reveals that the running time of PRMeth increased as the number of samples and features gradually increased, but there was no clear pattern in its memory usage (Additional file 1: Table S2).

### Evaluation of different methods using Zhang data

**Fig. 4:**
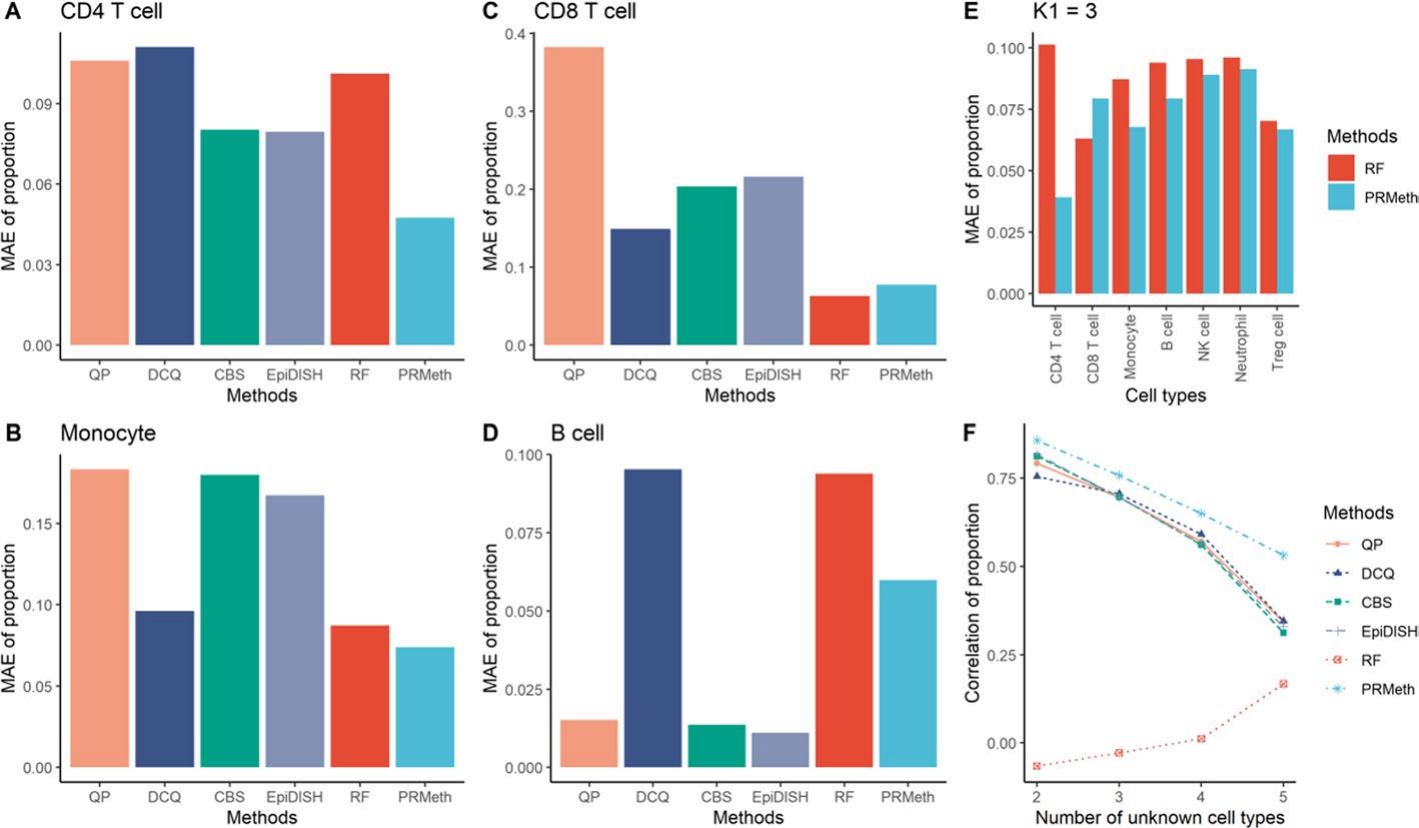
Accuracy of cell type proportions estimated by different methods. **A**–**D** The MAE between the true and predicted proportions of CD4 + T cells (**A**), monocytes (**B**), CD8 + T cells (**C**), or B cells (**D**) by the six methods when the number of known cell types is 4. **E** The MAE between the true and predicted proportions of each of all cell types by PRMeth and RF when the number of known cell types is 3. **F** The PCC between the true and predicted proportions of known cell types by the six methods at different numbers of unknown cell types

We then evaluated different methods on the Zhang dataset from three aspects, i.e., the accuracies of six methods in estimating the proportions of known cell types, the accuracies of PRMeth and RF in estimating the proportions of all cell types, and the overall performance of proportion estimates at different numbers of unknown cell types. First, by setting ${K}_{1}$ as 4, we calculated the MAE between the true and predicted proportions of each of the four cell types using the six methods. Figure 4A, B demonstrate that PRMeth had the lowest MAE at both CD4 + T cells and monocytes compared to other methods. Figure 4C shows that RF had the lowest bias (${MAE}_{RF}=0.0631$) at CD8 + T cells, followed by PRMeth (${MAE}_{PRMeth}=0.0775$). About the MAE of B cells, PRMeth ranked fourth, which was slightly higher than EpiDISH, CBS, and QP (Fig. 4D). In general, PRMeth had better results for the proportion estimates of a single cell type compared to other methods. A similar performance was obtained by PRMeth when ${K}_{1}=2, 3, 5$ (Additional file 1: Figures S4, S5 and S6). Second, we obtained the MAE between the true and predicted proportions for each of all cell types using PRMeth and RF when ${K}_{1}=3$. Except for CD8 + T cells, the MAE of PRMeth was lower than RF for the remaining six cell types (Fig. 4E). Overall, our method had higher accuracy in predicting the proportions of each cell type compared to RF when ${K}_{1}=2, \mathrm{3,4}, 5$ (Fig. 4E and Additional file 1: Figure S7). Finally, the PCC between the true and predicted proportions of known cell types obtained by the six methods at different numbers of unknown cell types is shown in Fig. 4F. As the number of unknown cell types increased, the PCC of both PRMeth and reference-based methods decreased. An exception is RF, which does not require reference data as input. It is clear that the PCC of PRMeth was always the highest and that of the reference-free method was always the lowest. When calculating the MAE between the true and predicted proportions of known cell types using the six methods at different numbers of unknown cell types, it is found that PRMeth consistently showed superiority over other methods (Additional file 1: Figure S8).

In addition, we estimated the methylation profiles of cell types using PRMeth and RF. We found that the accuracy and correlation of the methylation profiles obtained by PRMeth at different numbers of unknown cell types were higher than RF (Additional file 1: Figure S9).

### Evaluation of different methods using whole blood data

**Fig. 5:**
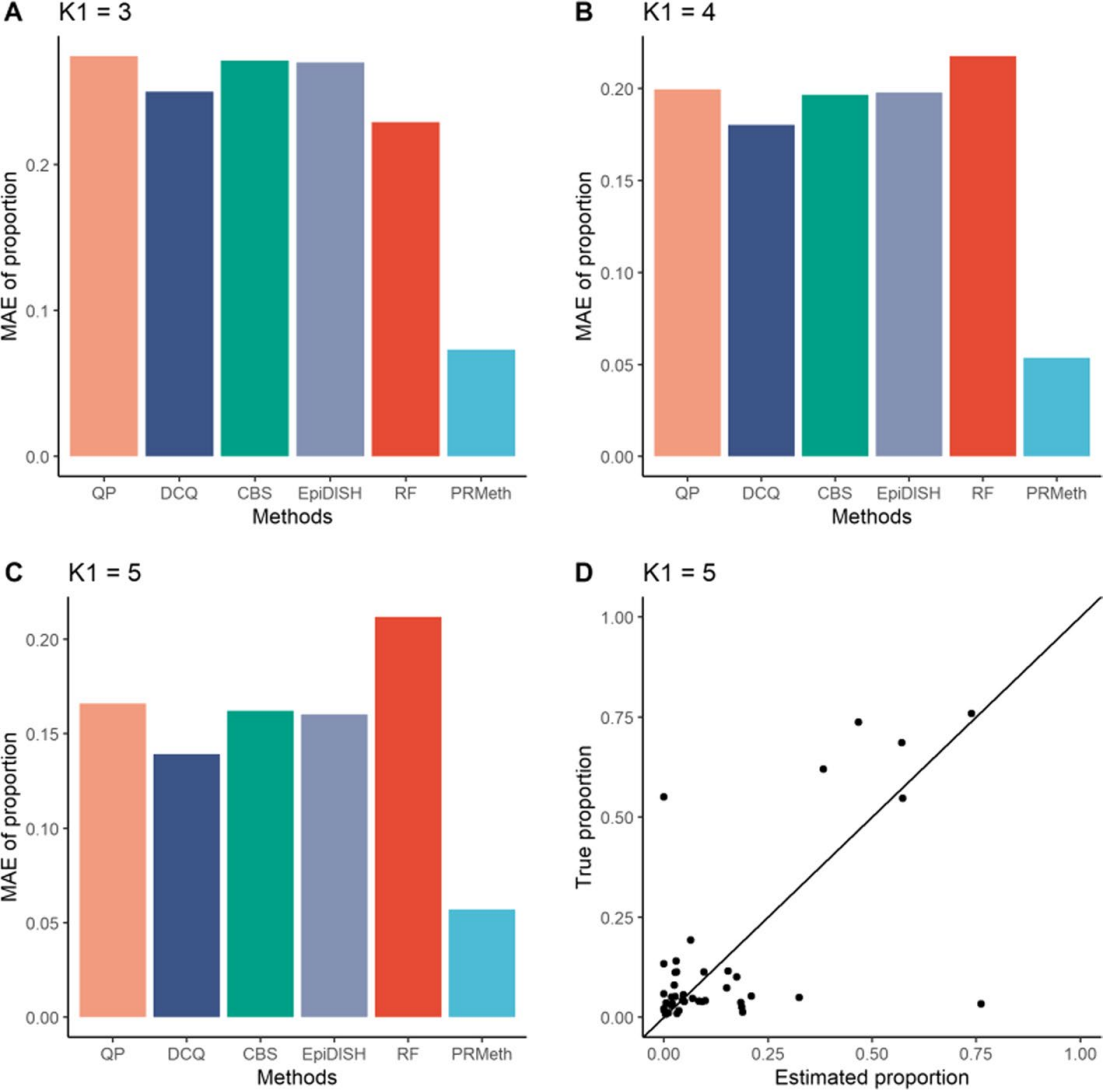
Accuracy of the estimated cell type proportions at different numbers of known cell types. **A**–**C** The MAE between the true and predicted proportions of known cell types by the six methods from whole blood samples with ${K}_{1}=3$(**A**), 4 (**B**), or 5 (**C**). **D** The estimation accuracy of the proportions of all cell types obtained by PRMeth at ${K}_{1}=5$

Next, we further validated our method on whole blood samples. We calculated MAE between the true and estimated proportions of known cell types by the six methods at ${K}_{1}=2, 3, 4, 5$. As shown in Fig. 5A–C, and Additional file 1: Figure S10, PRMeth showed the lowest bias at all values of ${K}_{1}$. We then compared all cell type proportions predicted by PRMeth with the true proportions measured by flow cytometry. This reveals that the estimation accuracy of PRMeth increased with increasing ${K}_{1}$ (Additional file 1: Figure S11 and Fig. 5D) and only a few predictions deviated from the true values at ${K}_{1}=5$ (Fig. 5D).

Similarly, we also estimated the cell type methylation profiles and found that the accuracy and correlation of PRMeth were consistently higher than RF (Additional file 1: Figure S12).

### Application to TCGA data

**Fig. 6:**
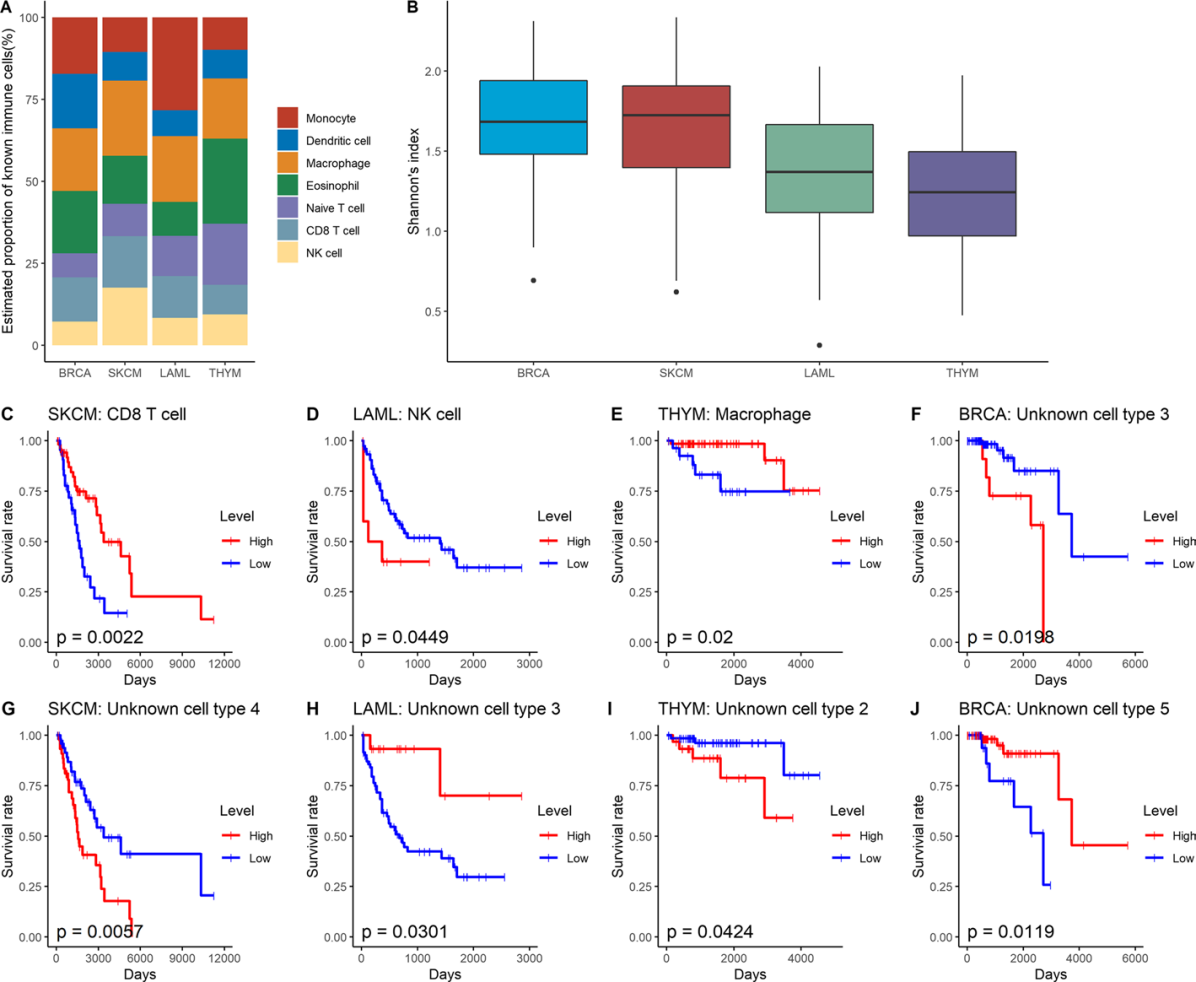
Application of PRMeth on TCGA dataset. **A** Relative proportions of seven types of immune cells in BRCA, SKCM, LAML, or THYM. **B** Shannon index of the four tumors. **C**–**J** Kaplan–Meier survival curves for SKCM stratified by abundances of CD8 + T cells (**C**) and unknown cell type 4 (**G**), LAML stratified by abundances of NK cells (**D**) and unknown cell type 3 (**H**), THYM stratified by abundances of Macrophages (**E**) and unknown cell type 2 (**I**), and BRCA stratified by abundances of unknown cell type 3 (**F**) and unknown cell type 5 (**J**). Using the *surv_cutpoint* function in the *survminer* package of R to divide cancer patients into high- and low-infiltrating groups based on the proportions of specific cell types. *p* values are obtained by the Log-rank test

Finally, we applied PRMeth to real tumor samples from TCGA. We selected seven types of immune cells (including monocytes, dendritic cells, macrophages, eosinophils, naive T cells, CD8 + T cells, and natural killer cells) as known partial reference data, and then deconvolved 400 tumor samples including 100 SKCM samples, 100 BRCA samples, 100 LAML samples, and 100 THYM samples. We first determined the total number of cell types in the four types of tumor samples using $\lambda \_BIC$ and the $K$ were 32, 29, 24, and 22, respectively. Because tumor tissue is a mixture of different cell types with a laminated structure that contains multiple cell types with different morphologies in each layer [38], we combined some cell types and assumed that the total numbers of cell types were 18, 16, 12, and 11 for SKCM, BRCA, LAML, and THYM, respectively. We then estimated the proportions of all cell types in these tumor samples using PRMeth and converted the absolute proportions of immune cells into relative proportions of each immune cell to all immune cells. As expected, different tumor samples showed different infiltration patterns of immune cells (Fig. 6A). In invasive breast carcinoma samples, macrophages occupied the highest proportion among all immune cells, which was consistent with previous literature findings [39] that a hallmark of breast cancer is high infiltration of M2 tumor-associated macrophages. The high infiltration levels of CD8 + T cells and macrophages in skin cutaneous melanoma samples were consistent with the study [40]. Acute myeloid leukemia and thymoma samples had high proportions of monocytes [35] and naive T cells [41], respectively.

To investigate the relationship between cell type proportions and tumor types, we used the Shannon index [42] representing the diversity of biomes to describe the heterogeneity degree of tumor samples. As shown in Fig. 6B, the heterogeneity scores (i.e., 1.6807, 1.6555, 1.3524, and 1.2401) of BRCA, SKCM, LAML, and THYM were significantly different, which illustrates the estimated proportions from PRMeth met the biological significance. We also analyzed the impact of the predicted proportions of cell types on the survival of cancer patients. We first used the *surv_cutpoint* function in the *survminer* package of R to divide cancer patients into high- and low-infiltrating groups based on the proportions of specific cell types (including known immune cells and estimated unknown cells), and then used Cox proportional hazards regression to calculate the survival rates of these two groups. We found that SKCM patients with a high infiltration level of CD8 + T cells and THYM patients with a high infiltration level of macrophages both had good overall survival (*p* = 0.0022 and 0.02, Fig. 6C, E), which was consistent with previous findings by Ma et al. [40] and Yang et al. [43]. In contrast, LAML patients with a high infiltration level of NK cells had poorer overall survival than those with a low infiltration level (*p* = 0.0449, Fig. 6D), which was consistent with the study's results [44] that the NK cells activated with high expression were associated with a poor prognosis. In addition, we also found that several unknown cell types had an impact on the survival of cancer patients (Fig. 6F–J).

## Discussion

In this paper, we proposed a cell type decomposition model (PRMeth) based on partially available DNA methylation data, which employs a non-negative matrix factorization and an iterative optimization algorithm. Given reasonable parameter settings, PRMeth could infer the proportions of all cell types and recover the methylation profiles of unknown cell types effectively. The study on the TCGA dataset showed that the immune cell proportions estimated by PRMeth were largely consistent with previous studies and met the biological significance. Compared to existing methods, the advantages of PRMeth are mainly reflected in the following points. First, PRMeth is applied to DNA methylation data that are relatively stable and easier to measure. Second, using partial DNA methylation data as a reference can reduce the difficulty of obtaining complete DNA methylation data. Third, PRMeth can infer not only the proportions of known cell types but also those of unknown cell types. Fourth, although the PRMeth method is driven by cancer research, it can be applied to other tissues, such as blood, to study the composition of cell types associated with other diseases, such as autoimmune diseases.

Despite its advantages, our study also suffers from the following limitations. First, our method requires the total number of cell types as input. The results on the Zhang dataset show that our method could obtain the exact total number of cell types using $\lambda \_BIC$. However, the total number of cell types is often uncertain because all cells of a complex tumor tissue form a laminated structure. In other words, cells are grouped by similarities so the total number of cell types can be determined by different groupings. Therefore, we encourage users to conduct downstream association analysis by choosing a reasonable $K$ in their study. Second, PRMeth does not apply to the estimation of cell type proportions for a single sample. In the future, we will expand the applicability of PRMeth and explore the relationship between cell type proportions and tumor subtypes, which may help to determine the optimal treatment regimen for a specific patient and predict potential targets for cancer immunotherapy.

## Conclusion

Different from the available reference-based and reference-free methods, the proposed method PRMeth is based on partial reference information, which is more in line with real clinical practice. It not only circumvents the difficulty of obtaining complete DNA methylation reference data but also obtains satisfactory deconvolution accuracy, which will be conducive to the reduction of medical costs, the analysis of tumor heterogeneity, and the exploration of new directions of cancer immunotherapy.

## Supplementary Information

**Additional file 1.** Supplementary Figures and Tables.

## Data Availability

The datasets used during the current study include the simulation dataset, the Zhang dataset, the whole blood dataset, and the TCGA dataset. The GEO accession codes for the simulation dataset are GSE88824 and GSE92843. The Zhang dataset can be downloaded from https://github.com/Hanyuz1996/EMeth. The whole blood dataset can be accessed through the GEO accession number GSE35069 and the link https://journals.plos.org/plosone/article?id=10.1371/journal.pone.0041361. The TCGA dataset is available via the Cancer Genome Atlas database and the GEO accession numbers GSE35069, GSE59250, and GSE71837. PRMeth is implemented in R and is freely available from GitHub (https://github.com/hedingqin/PRMeth).

## Abbreviations

TCGA: The cancer genome atlas
EWAS: Epigenome-wide association study
FFPE: Formalin-fixed paraffin-embedded tissues
BRCA: Invasive breast carcinoma
SKCM: Skin cutaneous melanoma
LAML: Acute myeloid leukemia
THYM: Thymoma
NK: Natural killer cell
NHBEC: Human bronchial epithelial cell
GEO: Gene expression omnibus
NSCLC: Non-small cell lung cancer
BIC: Bayesian information criterion
RF: Reference-Free
QP: Quadratic programming
CBS: CIBERSORT
DCQ: Digital cell quantification
EpiDISH: Epigenetic dissection of intra-sample heterogeneity
MAE: Mean absolute error
PCC: Pearson correlation coefficient
