# Supplementary material for: Deconvolution of tumor composition using partially available DNA methylation data
Source: BMC Bioinformatics. 2022 Aug 24;23:355. doi: 10.1186/s12859-022-04893-7 (PMC9400327; doi:10.1186/s12859-022-04893-7)
Supplement: Supplementary file 1 — Additional file 1. Supplementary Figures and Tables. [file 12859_2022_4893_MOESM1_ESM.docx]

**Deconvolution of tumor composition using partially available DNA methylation data**

**Supplementary Material**

**
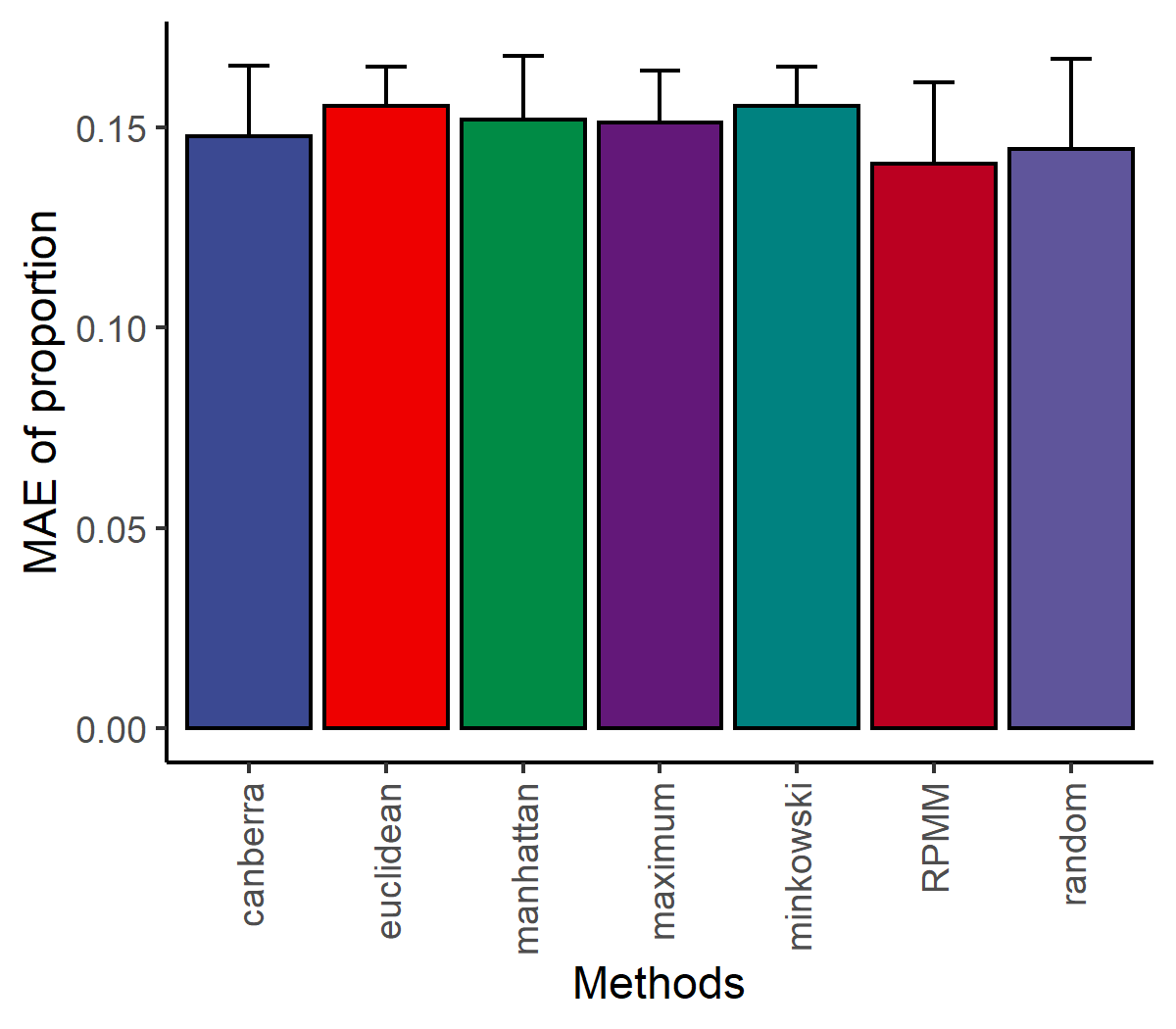
**

Supplementary Figure 1

Performance comparison of RPMM with different initialization approaches (including canberra, euclidean, manhattan, maximum, minkowski, and random). All simulations were repeated 20 times.


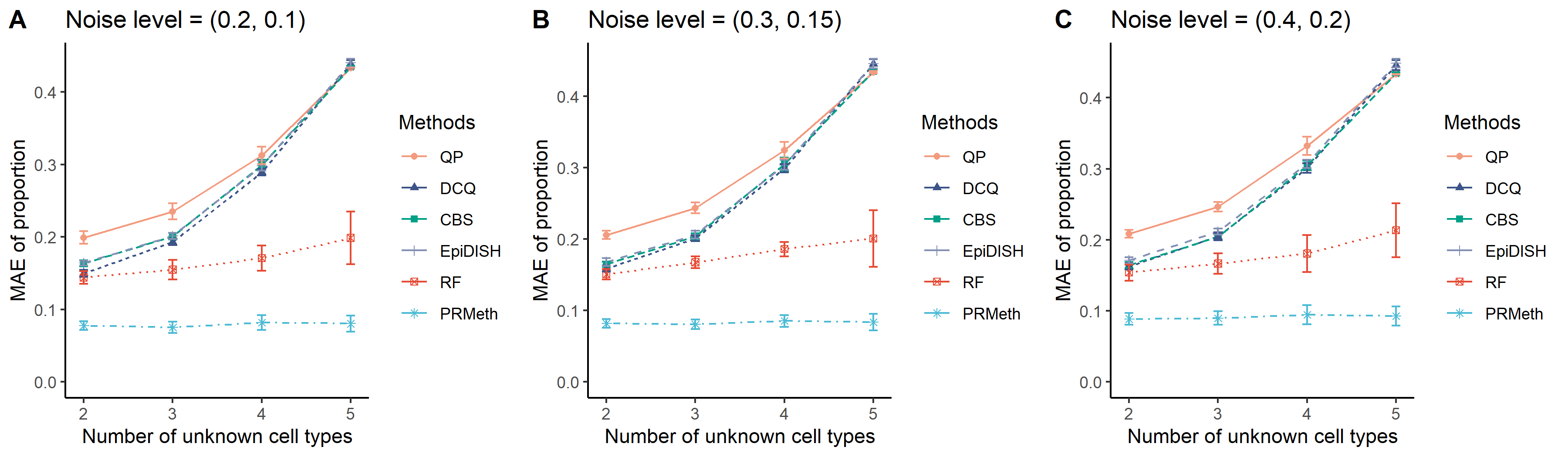


Supplementary Figure 2

The mean absolute error between the true and predicted cell type proportions obtained by six methods from the simulation dataset with different unknown cell type numbers for three noise levels (0.2, 0.1) (A), (0.3, 0.15) (B), and (0.4, 0.2) (C). All simulations were repeated 20 times.


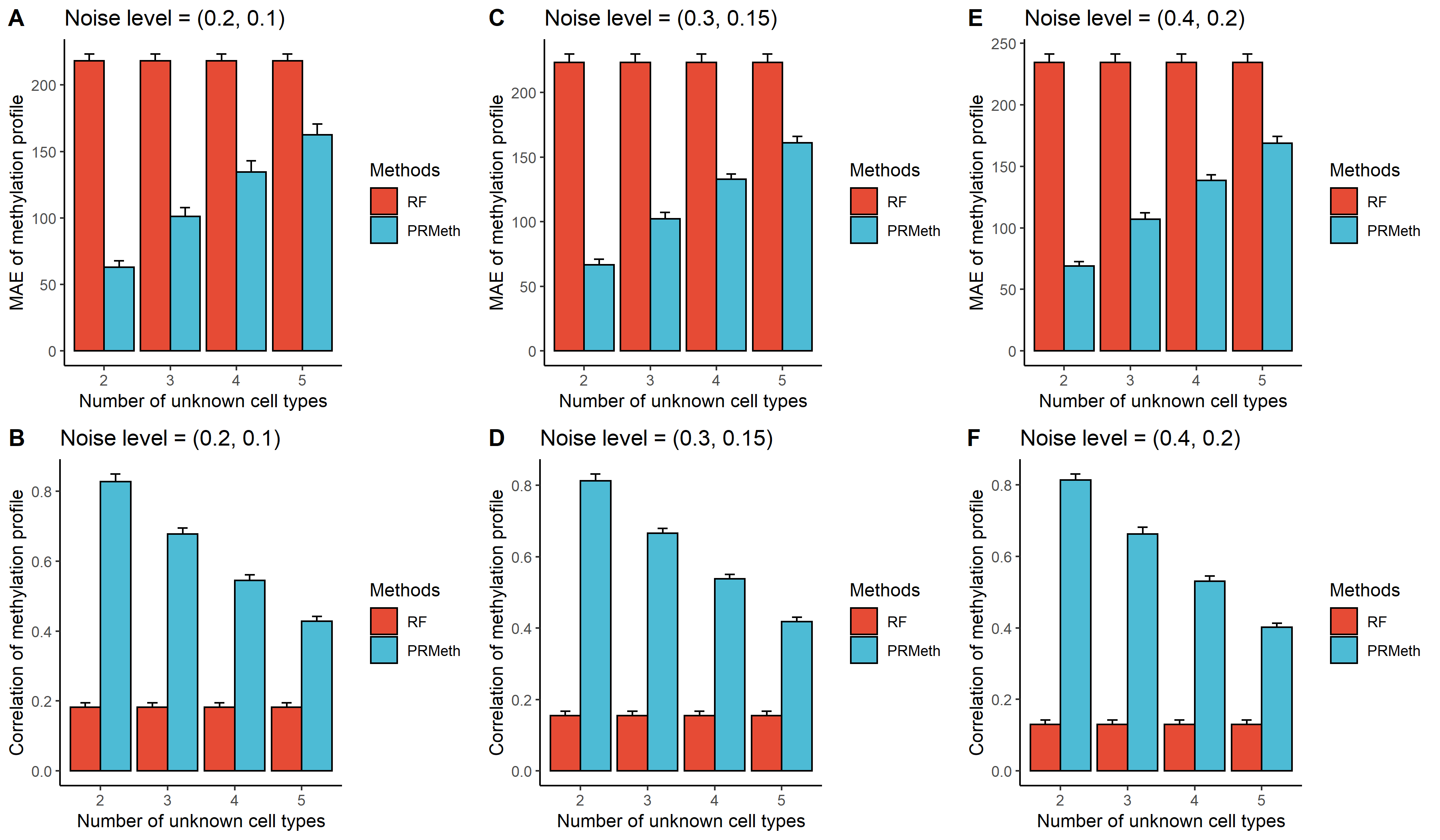


Supplementary Figure 3

The mean absolute errors and Pearson correlation coefficients between true and predicted cell type methylation profiles obtained by PRMeth and RF at different unknown cell type numbers for three noise levels (0.2, 0.1) (A-B), (0.3, 0.15) (C-D), and (0.4, 0.2) (E-F). All simulations were repeated 20 times.

| Supplementary Table 1 The average run time and average memory usage of six methods at 100 samples and 1000 CpG sites | | |
| --- | --- | --- |
| Methods | Time (s) | Memory (Mb) |
| QP | 0.02 | 26.20 |
| EpiDISH | 0.24 | 154.33 |
| DCQ | 1.16 | 136.41 |
| CBS | 11.02 | 269.3 |
| RF | 38.62 | 429.99 |
| PRMeth | 41.51 | 512.03 |
| All simulations were repeated 20 times. | | |

| Supplementary Table 2 The run time and memory usage of PRMeth | | | | | | | | | | |
| --- | --- | --- | --- | --- | --- | --- | --- | --- | --- | --- |
| Feature  Sample | 200 | | 400 | | 600 | | 800 | | 1000 | |
|  | Time (s) | Memory (Mb) | Time (s) | Memory (Mb) | Time (s) | Memory (Mb) | Time (s) | Memory (Mb) | Time (s) | Memory (Mb) |
| 20 | 5.69 | 455.29 | 10.55 | 344.38 | 15.56 | 457.83 | 20.25 | 354.82 | 25.64 | 256.21 |
| 40 | 6.69 | 745.40 | 12.65 | 826.81 | 19.13 | 658.11 | 24.40 | 488.06 | 30.07 | 306.19 |
| 60 | 8.08 | 745.39 | 14.70 | 554.73 | 21.62 | 669.54 | 27.47 | 1061.34 | 35.22 | 798.93 |
| 80 | 9.25 | 295.99 | 16.34 | 416.61 | 23.38 | 446.94 | 31.89 | 530.28 | 42.17 | 629.77 |
| 100 | 10.81 | 231.57 | 18.35 | 849.59 | 26.11 | 454.13 | 34.17 | 637.16 | 42.56 | 1115.71 |


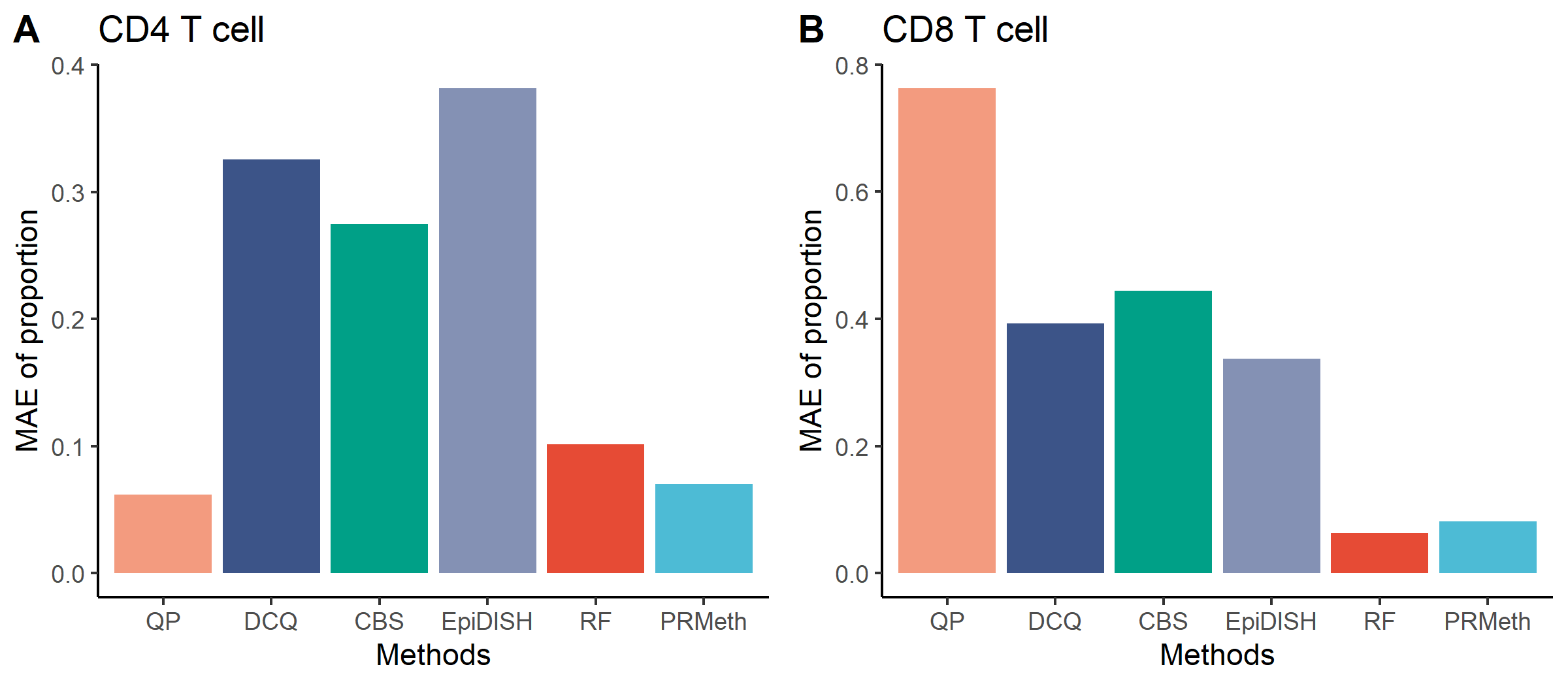


Supplementary Figure 4

The MAE between the true and predicted proportions of CD4+ T cells (A) or CD8+ T cells (B) by the six methods when the number of known cell types is 2.


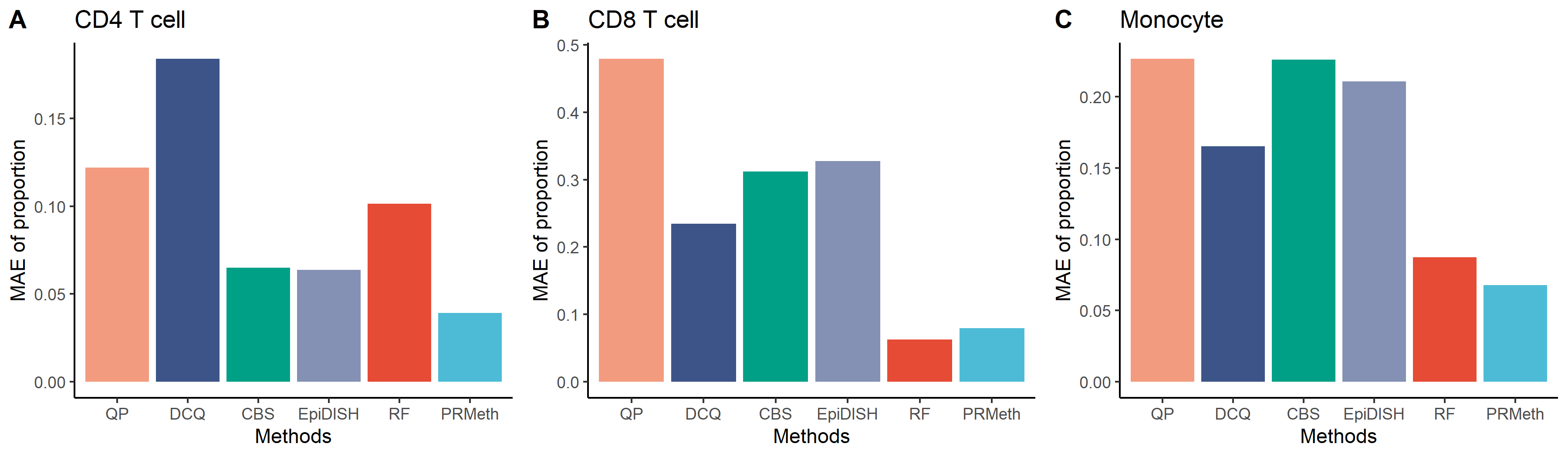


Supplementary Figure 5

The MAE between the true and predicted proportions of CD4+ T cells (A), CD8+ T cells (B), or monocytes (C) by the six methods when the number of known cell types is 3.


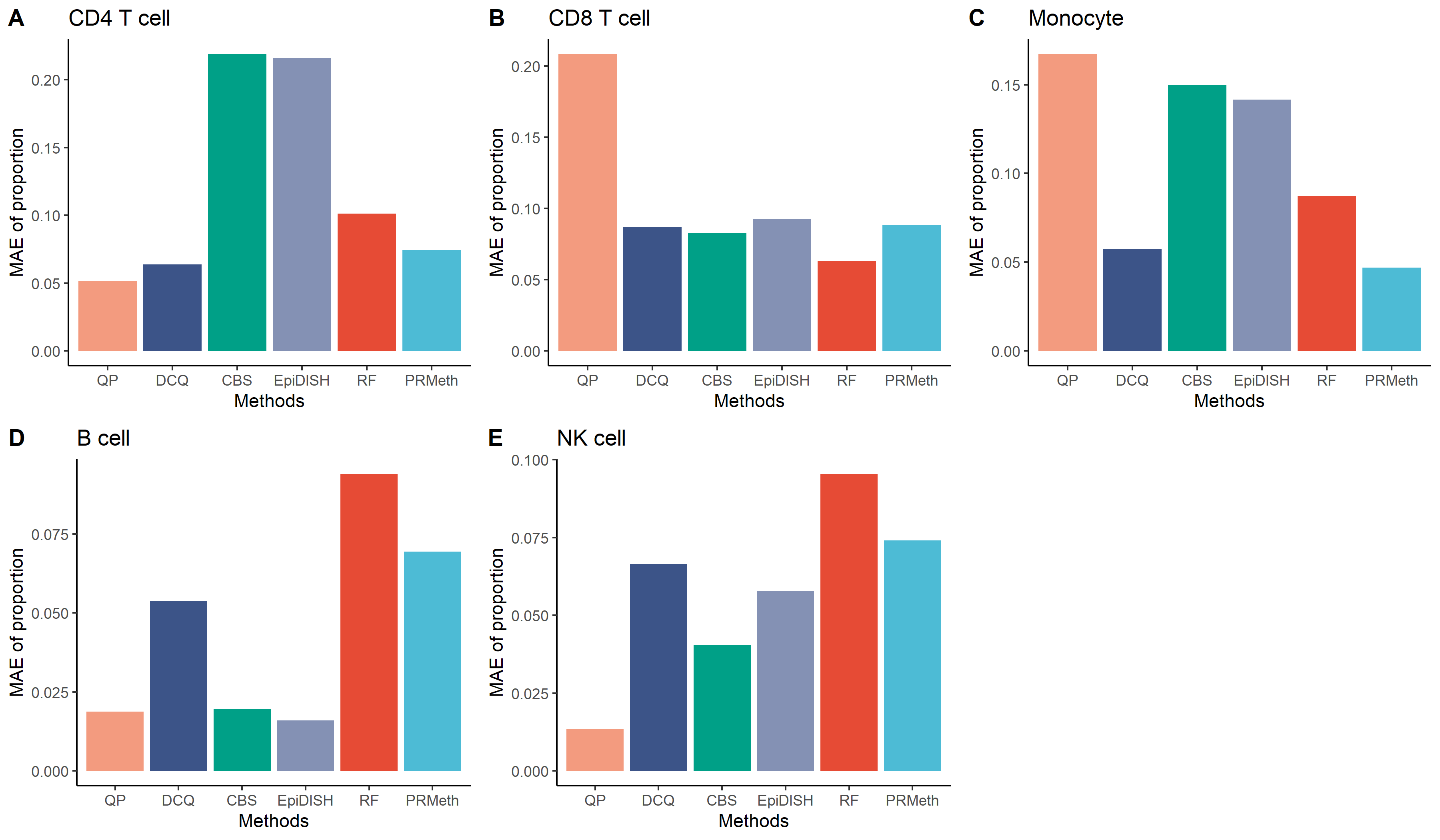


Supplementary Figure 6

The MAE between the true and predicted proportions of CD4+ T cells (A), CD8+ T cells (B), monocytes (C), B cells (D), or NK cells (E) by the six methods when the number of known cell types is 5.


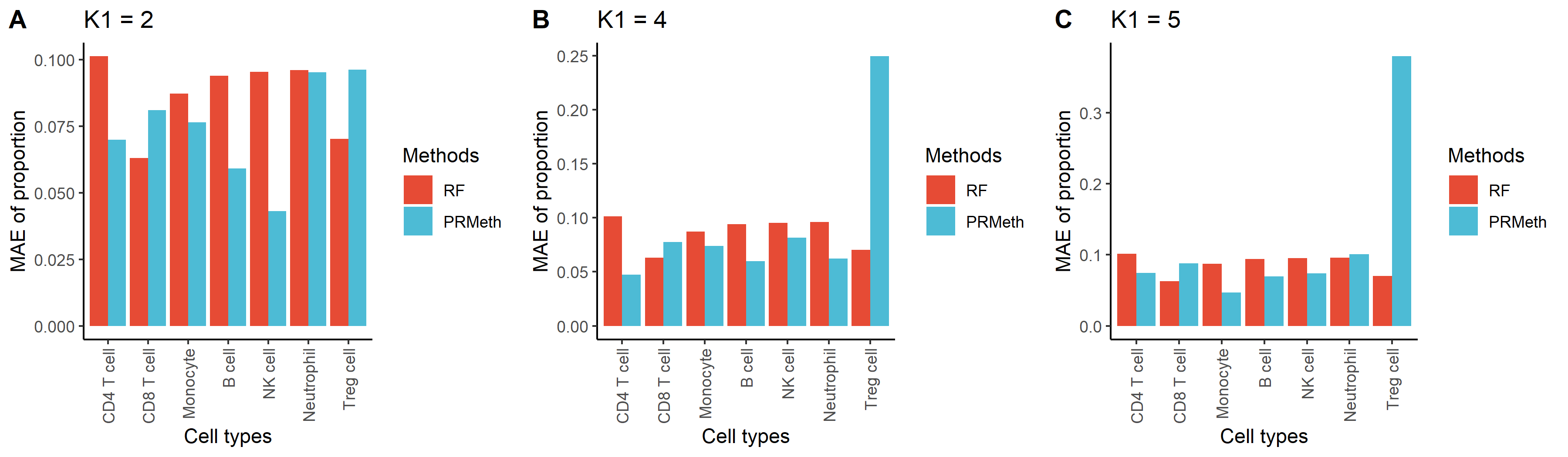


Supplementary Figure 7

The MAE between the true and predicted proportions of each of all cell types by PRMeth and RF when the number of known cell types is 2 (A), 4 (B), or 5 (C).


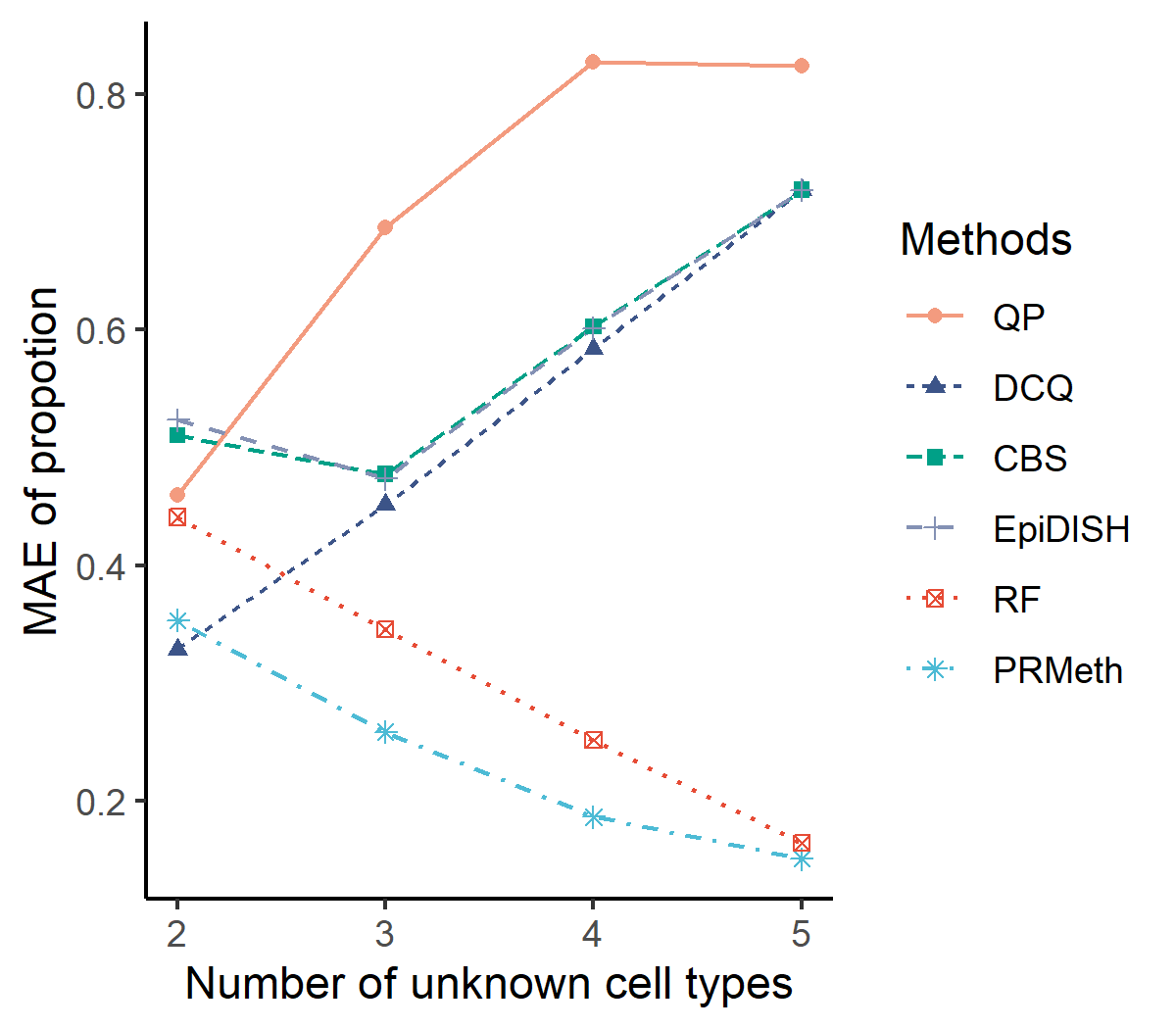


Supplementary Figure 8

The MAE between the true and predicted proportions of known cell types by the six methods at different numbers of unknown cell types.


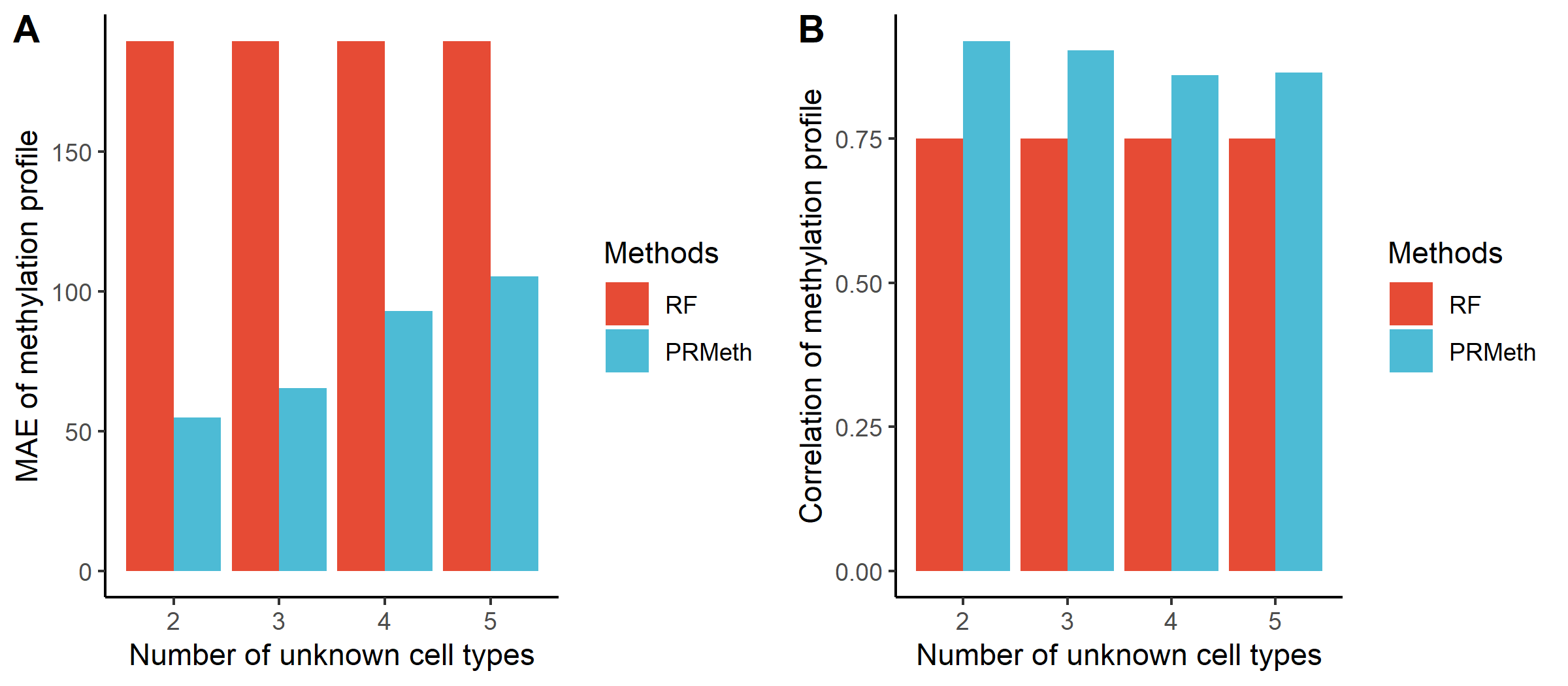


Supplementary Figure 9

The mean absolute errors (A) and Pearson correlation coefficients (B) between true and predicted cell type methylation profiles obtained by PRMeth and RF at different unknown cell type numbers.


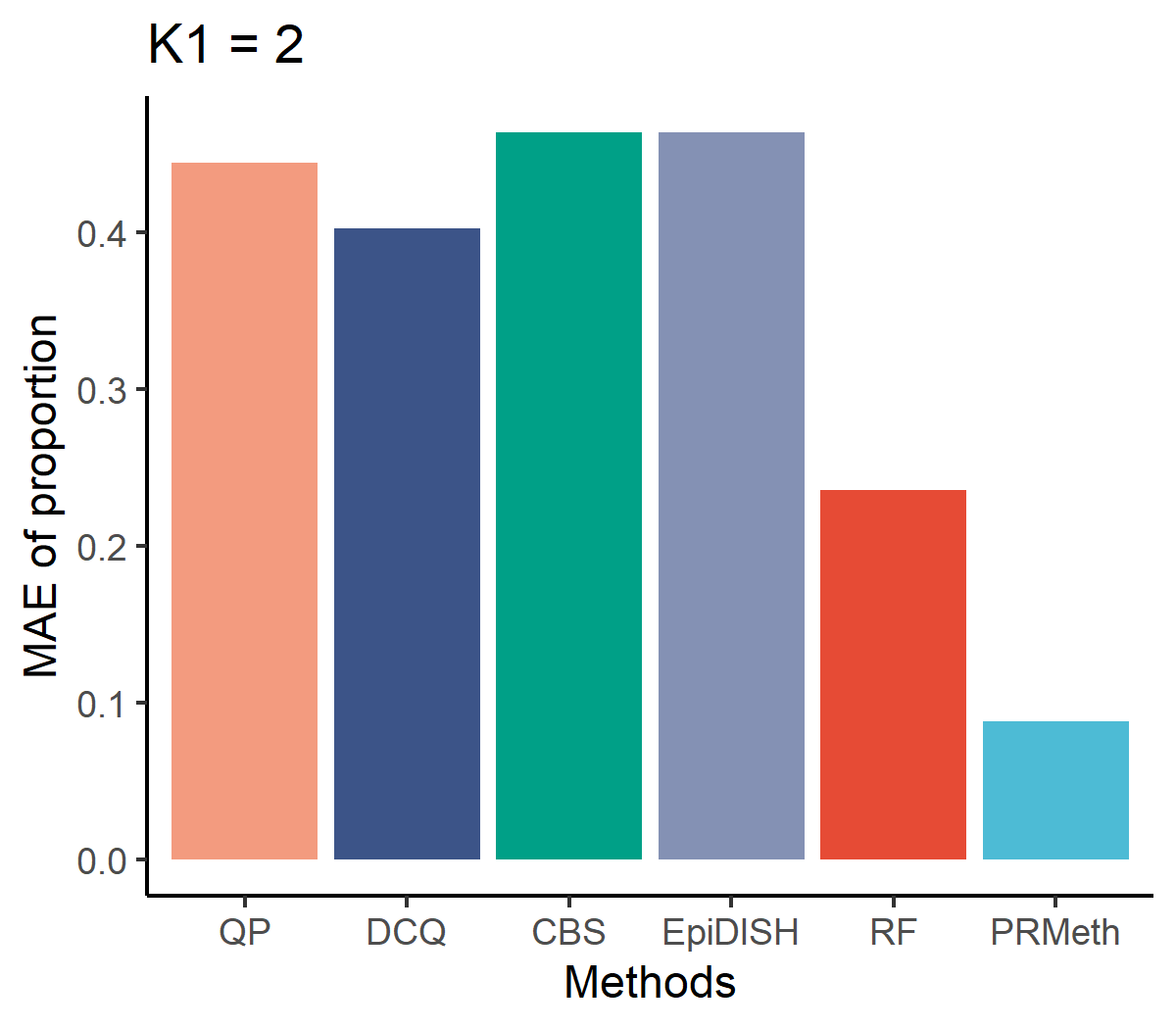


Supplementary Figure 10

The MAE between the true and predicted proportions of known cell types by the five methods from whole blood samples with $K_{1}=2$.


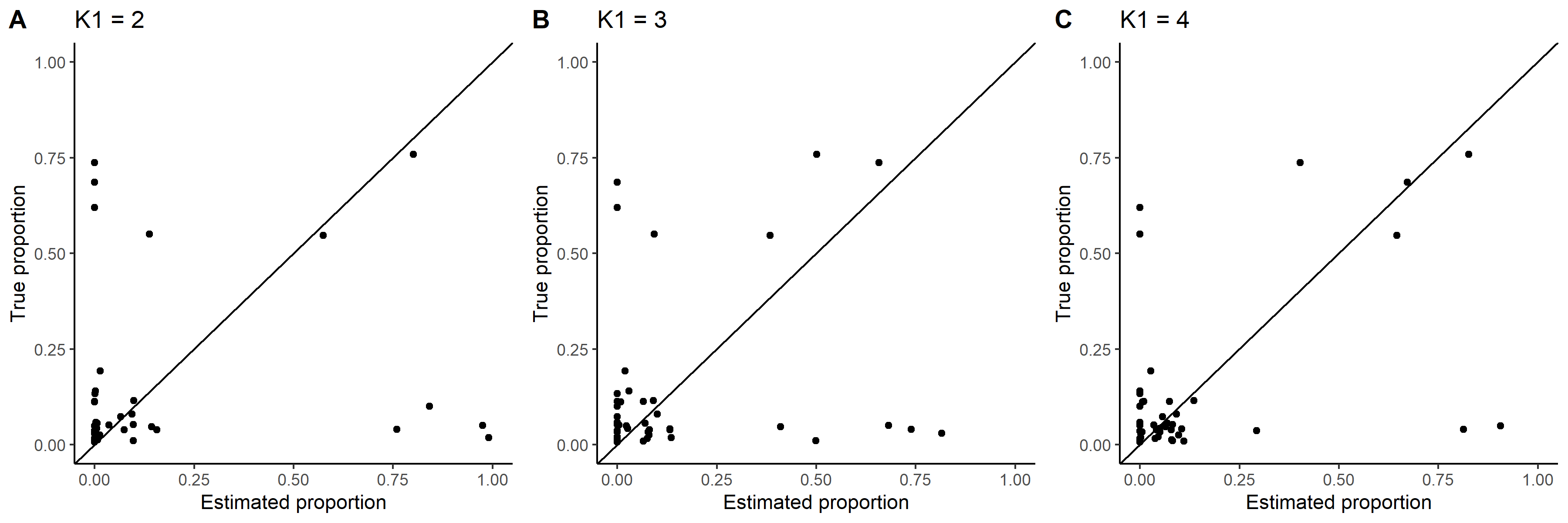


Supplementary Figure 11

The estimation accuracy of the proportions of all cell types obtained by PRMeth at $K_{1}=$ 2, 3, or 4.


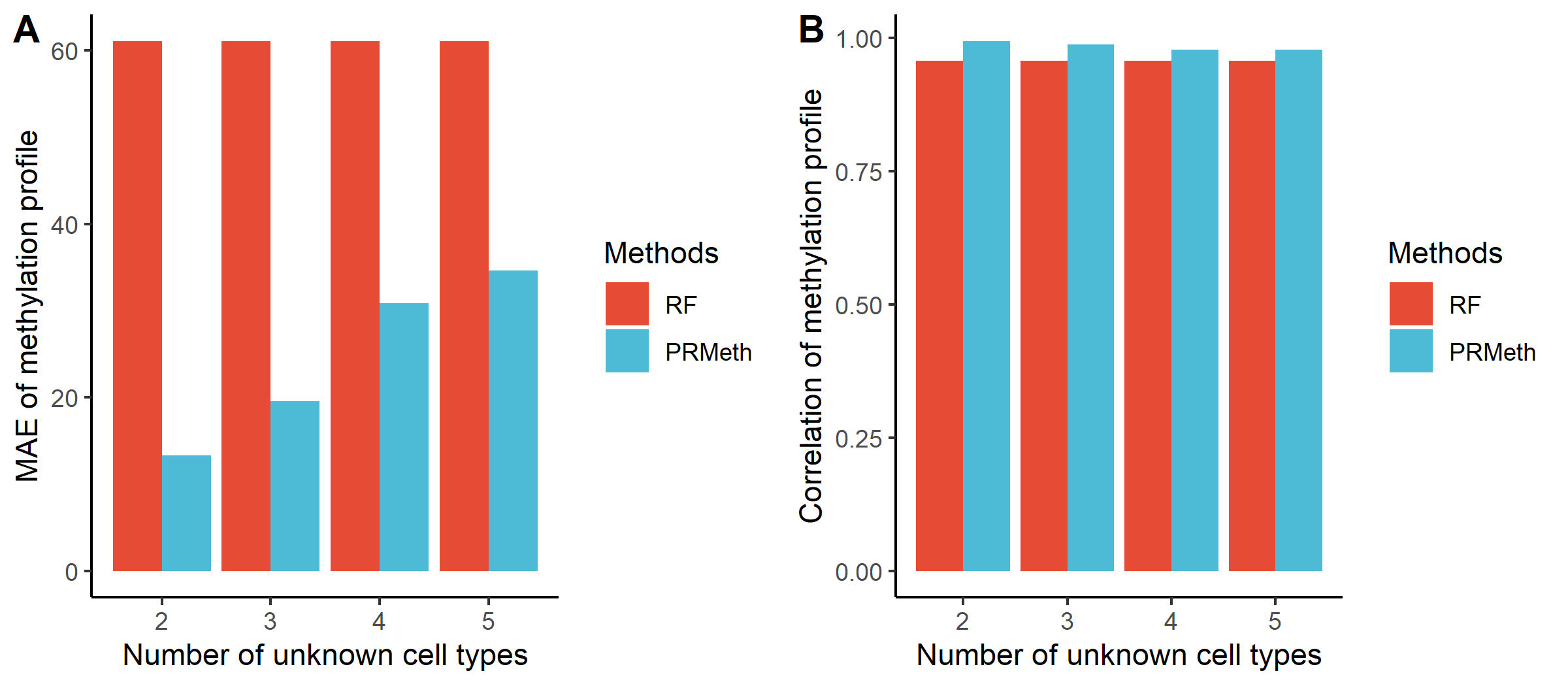


Supplementary Figure 12

The mean absolute errors (A) and Pearson correlation coefficients (B) between true and predicted cell type methylation profiles obtained by PRMeth and RF at different unknown cell type numbers.
